# Warpage Optimisation on the Moulded Part with Straight Drilled and Conformal Cooling Channels Using Response Surface Methodology (RSM), Glowworm Swarm Optimisation (GSO) and Genetic Algorithm (GA) Optimisation Approaches

**DOI:** 10.3390/ma14061326

**Published:** 2021-03-10

**Authors:** Mohd Hazwan Mohd Hanid, Shayfull Zamree Abd Rahim, Joanna Gondro, Safian Sharif, Mohd Mustafa Al Bakri Abdullah, Azlan Mohd Zain, Abdellah El-hadj Abdellah, Mohd Nasir Mat Saad, Jerzy J. Wysłocki, Marcin Nabiałek

**Affiliations:** 1 Faculty of Mechanical Engineering Technology, Universiti Malaysia Perlis, Arau 02600, Perlis, Malaysia; nasirsaad@unimap.edu.my; 2 Center of Excellence Geopolymer and Green Technology (CEGeoGTech), Universiti Malaysia Perlis, Kangar 01000, Perlis, Malaysia; mustafa_albakri@unimap.edu.my; 3 Department of Physics, Częstochowa University of Technology, 42-200 Częstochowa, Poland; joanna.gondro@pcz.pl (J.G.); wyslocki.jerzy@wip.pcz.pl (J.J.W.); nabialek.marcin@wip.pcz.pl (M.N.); 4 Faculty of Engineering, Universiti Teknologi Malaysia, UTM Skudai 81310, Johor, Malaysia; safian@utm.my (S.S.); azlanmz@utm.my (A.M.Z.); 5 Faculty of Chemical Engineering Technology, Universiti Malaysia Perlis, Kangar 01000, Perlis, Malaysia; 6 Laboratory of Mechanics, Physics and Mathematical Modelling (LMP2M), University of Medea, Medea 26000, Algeria; lmp2m_cum@yahoo.fr

**Keywords:** injection moulding process, straight drilled cooling channels, conformal cooling channels, Response Surface Methodology (RSM), Glowworm Swarm Optimisation (GSO), Genetic Algorithm (GA)

## Abstract

It is quite challenging to control both quality and productivity of products produced using injection molding process. Although many previous researchers have used different types of optimisation approaches to obtain the best configuration of parameters setting to control the quality of the molded part, optimisation approaches in maximising the performance of cooling channels to enhance the process productivity by decreasing the mould cycle time remain lacking. In this study, optimisation approaches namely Response Surface Methodology (RSM), Genetic Algorithm (GA) and Glowworm Swarm Optimisation (GSO) were employed on front panel housing moulded using Acrylonitrile Butadiene Styrene (ABS). Each optimisation method was analysed for both straight drilled and Milled Groove Square Shape (MGSS) conformal cooling channel moulds. Results from experimental works showed that, the performance of MGSS conformal cooling channels could be enhanced by employing the optimisation approach. Therefore, this research provides useful scientific knowledge and an alternative solution for the plastic injection moulding industry to improve the quality of moulded parts in terms of deformation using the proposed optimisation approaches in the used of conformal cooling channels mould.

## 1. Introduction

The injection moulding process is fast, consisting of an autonomous operation and consistent duplication quality. It is widely used in today’s modern moulding industry [1]. It comprises four main phases: filling, packaging, cooling and ejection. Nowadays, it has become more difficult for the moulding industry to produce high quality moulded parts while at the same time increasing the productivity rate, especially for small sized products which are complex in shape [2]. Warpage is one of the common defects in the injection moulding process which is difficult to eliminate [3,4]. It could occur due to an uneven temperature field which leads to the uneven rate of molecules that cool and shrink. It may happen because of the complexity of product design and several other influencing factors during the moulding process, such as moulding temperature, melting temperature, type of plastic material used, injection and packing pressure, packing and cooling time and definitely ambient temperature [5]. This type of defect results in uneven clearance or malfunction during the assembly process [2]. However, it can be reduced by properly setting the processing parameters [6]. So far, most mould making companies have relied on the best and guess approach to seek for an ideal setting of processing parameters in reducing warpage. This conventional technique may consume time and cost [7]. Today, with advances in computer technology and simulation software, the optimisation approach could be utilised in determining an optimum setting of processing parameters in a faster way with appropriate accuracy [8]. Therefore, many researchers have proposed numerous optimisation proposals to enhance the quality of moulded parts [9,10,11,12,13]. 

For instance, Chen et al. [9], introduced the systematic two stage optimisation approaches consisting of Taguchi’s method with an aid of Response Surface Methodology (RSM) and Genetic Algorithms (GA). This research also utilized the hybrid Genetic Algorithms with Particle Swarm Optimisation (GA-PSO) in determining the best length and minimising moulded part warpage. The specimen was moulded from PBT-2100 plastic material. Taguchi’s optimisation approach was employed to construct a set of Design of Experiment (DOE). By using ANOVA, significant parameters contributing towards length quality and warpage condition were identified. RSM was carried out as a first stage optimisation work. Then, hybrid GA-PSO was utilised as a second stage optimisation to solve the multi-objective parameter setting. Based on the two stage optimisation results, best length obtained was 170.483 mm which is closest to the drawing specification (170.5 mm). Warpage results have also been improved from 0.092 to 0.025 mm. However, this study only focuses on the conventional straight drilled cooling channels.

Oliaei et al. [10], proposed the Taguchi’s method with an aid of the artificial neural network (ANN) to minimise warpage and shrinkage of plastic spoon. The specimen was moulded from three types of materials which are Polylactic acid (PLA), blended PLA with thermoplastic polyurethane (TPU) and blended PLA with thermoplastic starch (TPS). A simulation using Autodesk Moldflow software was carried out to predict the specimen warpage and shrinkage condition. Next, DOE was constructed using Taguchi’s method and tested by simulation work to determine the warpage and shrinkage results for each DOE ran. By using ANOVA, the significant parameters affecting warpage and shrinkage condition were defined. Lastly, ANN was established to obtain the optimised results. Based on the ANN optimised result, warpage conditions are 1.367 mm for PLA, 0.4647 mm for PLA-TPU and 0.6928 mm for PLA-TPS. On the other hand, shrinkage conditions are 7.025% for PLA, 1.791% for PLA-TPU, and 2.728% for PLA-TPS. However, this study only focuses on the application of optimisation on different types of materials as well as the conventional straight drilled cooling channels mould without verification of experimental work.

Zhao et al. [11] utilised the sequential of Improved Efficient Global Optimisation (IEGO) algorithm. In this research the Non-Dominated Sorting-Based Genetic Algorithm II (NSGA-II) optimisation method has also been carried out. The objective was to improve the quality of the moulded front shell of LCD TV moulded from ABS AF303 material. The quality of this specimen was measured in terms of warpage, shrinkage, and sink marks conditions. Firstly, DOE was constructed using Optimal Latin Hypercube sampling. Next, each DOE result was determined using Moldflow analysis. Then, the mathematical model was obtained by utilising the Kriging surrogate model. This mathematical model was then used in IEGO and NSGA-II to obtain the optimised results. Based on the IEGO results, warpage was reduced by 11.13%, shrinkage was reduced by 20.14% and sink mark was reduced by decreasing the melt temperature and increasing the injection time setting. Meanwhile, for NSGA-II optimised result, warpage was reduced by 14.66%, shrinkage was reduced by 13.14% and no significant change was observed on sink mark results. As previously mentioned, this study only focuses on the application of straight drilled cooling channels mould.

From the review that has been done, the quality of the moulded parts can be improved by employing an optimisation approach [9,10,11]. Nevertheless, the optimisation application in previous studies only focused on straight drilled cooling channels mould application [9,10,11]. Therefore, the use of optimisation in the conformal cooling channels is still missing. To date, several researchers have examined the advantages of conformal cooling channels. Most of the results showed enhanced efficiency of the moulded part produced [14,15,16]. 

This can be exemplified in Saifullah and Masood [16], where they introduced the Bi-Metallic Conformal Cooling Channels (BCCC). This cooling channel utilised a high thermal conductive copper tube insert (CTI). Furthermore, polypropylene (PP) was used to mould plastic canisters. ANSYS and Moldflow software were utilised to simulate the thermal structure on the injection mould. From the simulation results, it was reported that, BCCC with 2 mm CTI provides a superior design option compared to the conventional straight cooling channels (CSCC). Then, experimental works were performed and results from the experiment indicated that, the cooling stage duration is lesser compared to the CSCC. Temperature variation was reduced from 10 °C to 8 °C and cooling time was reduced by 35% (from 8 s to 5 s) using BCCC as compared to CSCC.

Park and Dang [15], proposed the use of insert with spiral conformal cooling channels to cool thicker walls area. This cooling channel was developed using selective laser melting (SLM) technology. A car door panel with varying thickness was used as a moulded part specimen. To determine the heat distribution on the moulded part and cavity surface, 3D modelling and formulation of cooling process were established. Then, conformal cooling channels were designed with verification of Moldflow analysis. Based on the simulation results, the spiral conformal cooling channel provides better temperature distribution with average temperature which was lower as compared to the original cooling channels with baffles. In addition, cooling time was also reduced by 23% (from 39 to 30 s) when using this conformal cooling channel.

These previous studies proved that most of researchers only focused on designing the conformal cooling channels to reduce the cycle time duration per say [15,16]. However, the optimisation exploration in determining the optimal setting of processing parameters to optimise the part quality moulded by conformal cooling channels mould is still missing. 

On the other hand, the optimisation algorithm can be devided in two main categories which are swarm intelligent based (e.g., Glowworm Swarm Optimisation) and evalutionary algorithm based (e.g., Genetic Algorithm). The differences and purposes of these algorithms are as shown in Table 1.

**Figure 1:**
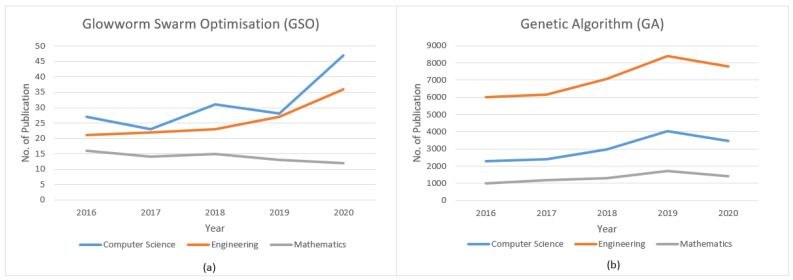
Studies for top three fields from 2016 to 2020 based on scopus database, (**a**) Glowworm Swarm Optimisation (GSO), (**b**) Genetic Algorithm (GA).

In addition, Genetic Algorithm (GA) is the optimisation approach that has been explored by many researchers in the engineering field [9,10,11,12,13]. On the contrary, Glowworm Swarm Optimisation (GSO) optimisation approach has gained lack of attention by previous researchers in engineering field. Figure 1 shows the studies using GSO and GA for top three fields from 2016 to 2020 based on the Scopus database.

In this study, Glowworm Swarm Optimisation (GSO) and Genatic Algorithm (GA) will be utilised on conventional straight drilled and Milled Groove Square Shape (MGSS) conformal cooling channels mould, while Response Surface Methodology (RSM) will be carried out to obtain the mathematical model as objective function for GSO and fitness function for GA. The main objective is minimising warpage on front panel housing moulded from ABS. DOE will be constructed via Full Factorial Design (FFD) based on selected processing parameters. It is then widened by adding a face-centered Central Composite Design (CCD), which increases the importance of the model with minimal DOE executions.

Then, Autodesk Moldflow Insight 2013 (AMI 2013) simulation software is carried out in obtaining warpage for each arrangement setting in DOE before the significant parameters that influence warpage on the moulded part were defined through Analysis of Variance (ANOVA). The mathematical model yielded was utilised in Glowworm Swarm Optimisation (GSO) and Genetic Algorithm (GA) optimisation approaches to decide on the best parameters setting which reduced warpage on the specimen produced by both straight drilled and MGSS cooling channels. After that, all parameters settings obtained from simulation analysis and each optimisation approach will be verified through an experimental work and the optimisations’ performances will be compared.

## 2. Methodology

**Figure 2:**
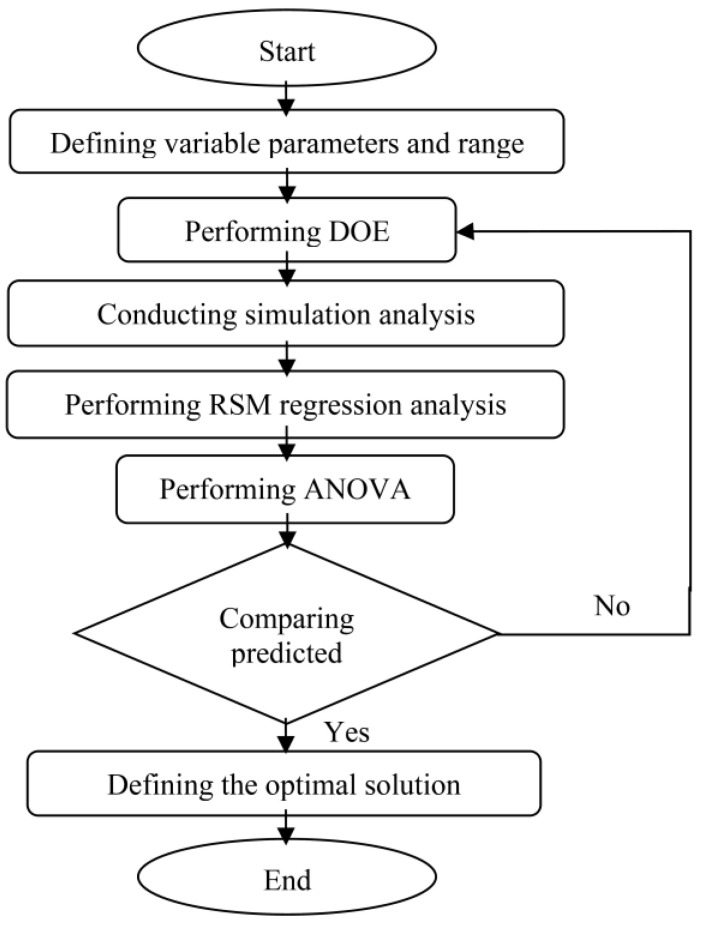
Response Surface Methodology (RSM) flowchart.

Response Surface Methodology (RSM) which uses statistical methods can be classified as a conventional optimisation method. Through RSM, the correlation between independent parameters and dependent response either on two or three-dimensional hyperbolic surfaces can be illustrated [17]. In addition, mathematical response function of second order polynomial model was obtained in this study and was carried out in GSO and GA optimisation approaches. The simulation studies collected essential data for the development of the response model [18,19]. The flowchart of RSM used in this study is presented in Figure 2.

### 2.1. DefiningVariable Parameter and Range

The temperature of coolant, cooling time, packing pressure and melting temperature were chosen as independent parameters in this study due to noteworthy impacts on the warpage condition [9,12,13]. The range of each parameter for cooling channels is presented in Table 2. Value of the range for each independent parameter was determined based on the recommended material processing parameters and simulation studies.

### 2.2. Performing DOE

Next, the Full Factorial Design (FFD) and the additional four center points were chosen to screen the model together with the main parameters that affect the warpage condition via Design Expert 7.0 software (Stat-Ease Inc., Minneapolis, MN, USA). By extending DOE using face centered CCD, the curvature of the tabulated warpage condition was analysed. Subsequently, 30 series with defined condition have been produced. All the DOE series were customary in AMI software to assess warpage values on the moulded part for both types of cooling channels.

### 2.3. Conducting Simulation Analysis

**Figure 3:**
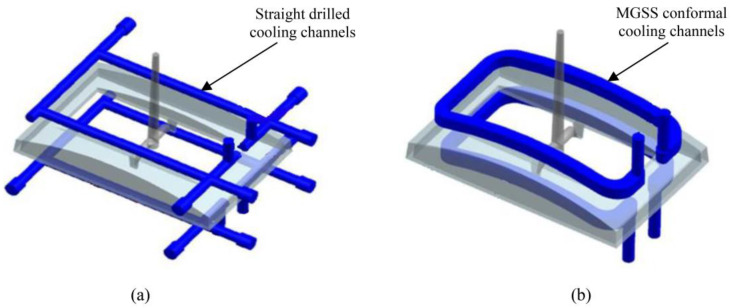
Front panel housing, (**a**) with straight drilled cooling channels, (**b**) with Milled Groove Square Shape (MGSS) conformal. cooling channels.

Injection moulding simulation analysis has been carried out via AMI 2013 (CAE software) to determine warpage state on front panel housing moulded for automotive application. It has an average thickness of 2.5 mm, moulded using ABS Toyolac 700-314 material grade produced by Toray Plastics (Malaysia) Sdn Bhd., Prai, Penang, Malaysia using straight drilled and MGSS cooling channels moulds as presented in Figure 3. ABS material was chosen due to its application in automotive industry which represents the current trend of moulded products in the market [20]. The 3D data of moulded part, gating system and cooling passages were designed and imported into AMI software (Autodesk Moldflow Insight 2012, Moldflow, Melbourne, Australia) to be meshed. To enhance the reliability of simulation results, the P20 steel mold insert and the 80 tonne injection molding (Nissei NEX1000, Nissei Plastic Industrial Co., LTD., Minamijo, Japan) specification were configured in the software. Afterwards, Cool (FEM) + Fill + Pack + Warp analysis was carried out to assess the warpage value for all series generated by DOE.

### 2.4. Performing RSM Regression Analysis

All warpage values determined by simulation studies were utilised in RSM regression analysis. The mathematical model that expresses the correlation between the independent parameters and the response will be obtained through regression analysis via Design Expert 7.0 software. This investigation was executed via second order polynomial regression model. 

### 2.5. Performing Analysis of Variance (ANOVA)

After that, the results were analysed via ANOVA to determine whether the regression models are statistically significant or vice versa by defining the probability value for both straight drilled and MGSS cooling channels. By executing the similar analysis, the main influencing parameters that affect the warpage condition will be identified.

## 3. Glowworm Swarm Optimisation

The GSO operators, also known as glowworms, carry an amount of luminescence called luciferin [21,22,23]. Each glowworm uses a proportional artificial luciferin to transfer the relevance of its present location by assessing its neighbours’ reliance on the objective function. The neighbourhood is decided based on the range of radial sensors. By selecting a neighbour via probabilistic component, each glowworm will move towards its neighbour which has much higher luciferin as it is attracted to neighbours who glow brighter than its own. In simple words, the glowworm will be attracted to the brighter neighbour. As a final point, the development of glowworms swarms formed depends on the nearby data and a specific neighbouring interaction to generate multiple multimodal function [21,23]. 

**Figure 4:**
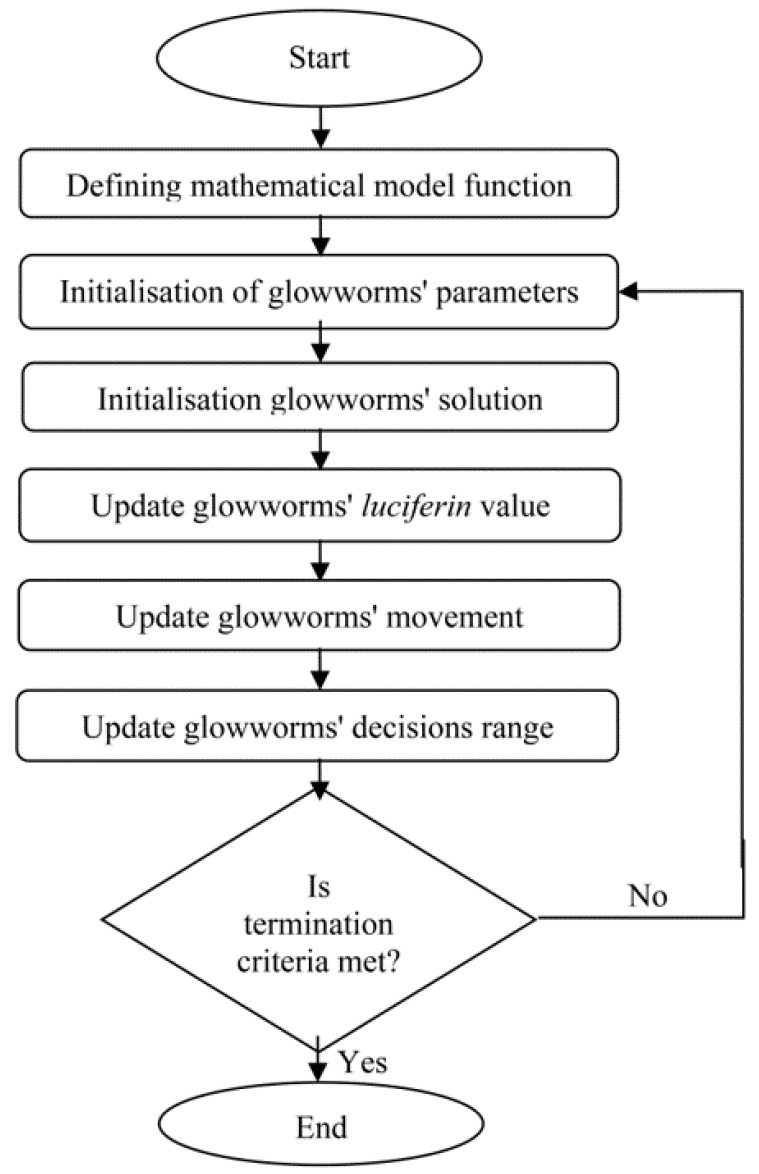
Flowchart of Glowworm Swarm Optimisation (GSO).

In this study, the GSO was used to optimise processing parameters of the moulding process for the two types of cooling channels based on the RSM mathematical models. MATLAB R2014a software was used to perform the GSO approach. The flowchart of GSO used in this study is presented in Figure 4.

### 3.1. Determining Mathematical Model Function Phase

Mathematical model generated in RSM analysis has been used as a model function in GSO analysis. Warpage has been set as formulated objective function to be reduced as much as possible.

### 3.2. Initialisation of Glowworms’ Parameters Phase

In this study, the injection moulding parameters were selected as initial glowworms’ parameters. The range of each parameter will enable the creation of the solution space in GSO analysis. At this point, the maximum iteration and initial glowworms population size have been set based on the conditions of this study to spread the GSO agents randomly in the solution space. For both cases of cooling channel designs, the number of glowworms and iteration used in this study has been set at 30 and 50 respectively.

### 3.3. Initialisation Glowworms’ Solution Phase

In the first step of the solution, the luciferin value and sensor range were the same for each glowworm. In this study, the initial luciferin value $\left(l_{o}\right)$, luciferin enhancement constant (γ), beta (β), step size (*s*), luciferin decay constant (ρ), neighborhood range, $r_{d}^{i}\left(t\right)$ and parameter used to manage the neighbours number $\left(n_{t}\right)$ were customised based on the basis of the optimal setting of GSO control parameter. Thus, the optimal setting of controlled parameters for both types of cooling channels design are: β=0.08, ρ=0.4, γ=0.6 and $r_{d}^{i}\left(t\right)=3$ [23].

### 3.4. Updated Glowworms’ Luciferin Value Phase

At the initial phase, the luciferin value of each glowworm was the same as previously mentioned. Subsequently, due to the predisposal by the current location objective function value for each luciferin, the value of luciferin has been modified. The luciferin will be modified based on Equation (1) to obtain the updated luciferin value;

(1) li(t+1)=(1−ρ)li(t)+γJi(t+1)

where, li(t) is the level of luciferin for glowworm i at time t, ρ is the decay constant for luciferin (0<ρ<1), and Ji(t) representing the objective function at agent i’s location at time t [23].

### 3.5. Updated Glowworms’ Movement Phase

In this phase, the probabilistic mechanism was used to move each glowworm that contained less luciferin value to its neighbour that has higher luciferin value. If agent *i* was moved towards agent *j*, the probability of movement can be estimated using Equation (2):

(2) $$P_{ij}\left(t\right)=\frac{l_{j}\left(t\right)-l_{i}\left(t\right)}{\sum_{k\in N_{i}\left(t\right)}l_{k}\left(t\right)-l_{i}\left(t\right)}$$

where, $l_{i}\left(t\right)$ is the luciferin value for glowworm i, d(i, j) is the Euclidean distance between agent *i* and *j* [22,23].

At that point, Equation (3) can be used to estimate the movement of glowworm *i*:

(3) $$x_{i}\left(t+1\right)=x_{i}\left(t\right)+s\left[\frac{x_{j}\left(t\right)-x_{i}\left(t\right)}{\left\|x_{j}\left(t\right)-x_{i}\left(t\right)\right\|}\right]$$

where *s* is the size of step [23].

### 3.6. Updated Glowworms’ Local Decisions Range Phase

From this updated glowworms’ local decisions range phase, a set of optimal variable parameter in injection moulding process which depends on the input range has been obtained to achieve minimal warpage condition. This phase was used in multimodal functional landscape to assess multiple peaks. In each iteration, the local decision domain must be updated when the number of neighbours changes by using the rule as expressed in Equation (4):

(4) $$r_{d}^{i}\left(t+1\right)=\min\{r_{s},\max\left\{0,r_{d}^{i}\left(t\right)+\beta (n_{t}-\left|N_{i}\left(t\right)\right|)\}\right\}$$

where $r_{d}^{i}\left(t+1\right)$ is glowworm local decision domain of i at iteration of t+1, β is the constant parameter (that affects neighbour domain change rates) and $n_{t}$ is the threshold (that controls the number of neighbours) [22,23]. 

## 4. Genetic Algorithm

Genetic Algorithm (GA) is a smart manipulation via random investigation utilized in optimization complications [24]. It replicates the nature opposition procedure where better fitness will take over the weaker fitness as mentioned in Darwin’s Theory [24]. In GA, selection, crossover and mutation are important operators that need to be given attention [24]. These operators can be divided into three phases. First, in the selection phase, the chromosomes are selected as parents. Next, new chromosomes have been created based on the combination of selected gene for both parents in the crossover phase. Afterwards, the chromosome’s bit number is flipped at random location in the mutation phase. Then, the chromosomes are evaluated using certain fitness criteria in order to select the best fitness and discharge the others. The optimum solution which is lowest warpage value is determined after the process is repeated until the best fitness chromosome was obtained.

**Figure 5:**
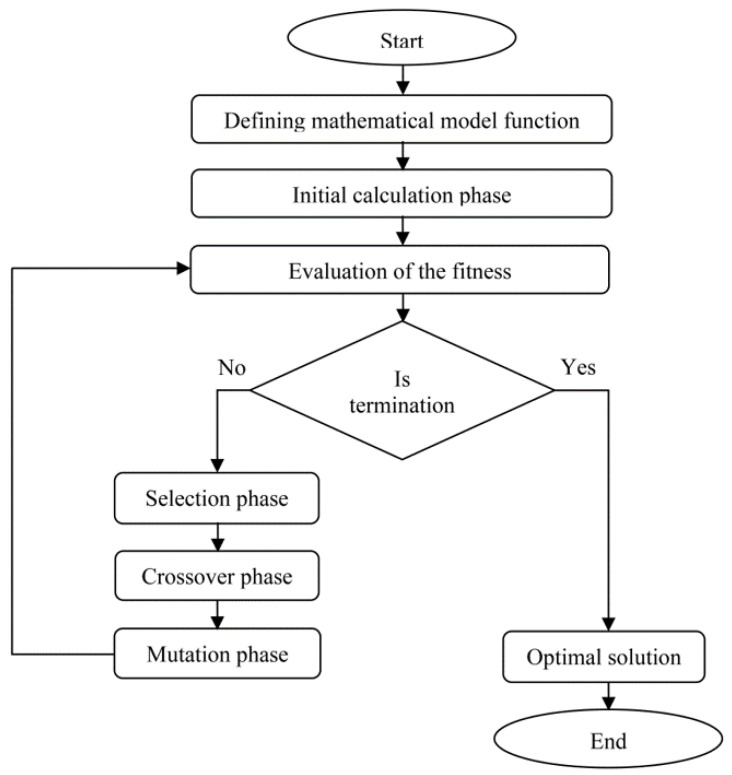
Flowchart of Genetic Algorithm (GA).

In this study, a set of optimal processing parameters in injection mouding process was obtained in order to minimise warpage of the moulded part using GA optimisation method. The result will be compared with RSM and GSO optimization methods. Second order RSM mathematical model was used in GA and GSO optimisation method. This type of optimisation approach is quite popular in injection moulding studies. Therefore, the performance of this optimisation approach will be investigated in this study. The analysis of GA was carried out using MATLAB R2014a software and the flowchart of GA is shown in Figure 5. 

### 4.1. Determining Mathematical Model Function

The mathematical model function used in GA analysis was obtained using the RSM analysis. This model has been used in GA as an objective function. Warpage of the moulded part has been taken as formulated objective function of this study. 

### 4.2. Initial Calculation Phase

In this stage, injection moulding parameters as well as the range of each parameters were set as input parameters for initial calculation in GA. The preliminary population size, generation number, crossover probability, mutation probability and bit number of each variable were set based on the condition of this study. The GA agents (which are chromosomes) will be created randomly in the solution space after each variable has been set. In this study, population size, number of generations, crossover probability and mutation probability have been set to 50, 23, 0.40 and 0.01 respectively [25]. Furthermore, the bit number for each variable has been set. 

### 4.3. Selection Phase

The first operator applied on each population occurs in this phase. During this phase, the parent will be built from chromosomes that are selected from the population. This parent will be applied for the crossover phase. The fitness of chromosomes was a criterion for chromosomes selection. The best chromosomes would be able to survive and generate new generation as mentioned in the Darwin Theory [25,26]. 

### 4.4. Crossover Phase

Crossover occurs throughout the entire evolution process based on crossover probability setting. In this phase, a new chromosome (offspring) has been produced based on the combination of selected genes from parent chromosomes. The goal of this phase is to generate a new chromosome. The new chromosome that has been developed should be better compared to their parents. The best characteristics of each parent will produce better chromosomes. 

### 4.5. Mutation Phase

In this phase, mutation will help the population to avoid becoming stagnant in the solution space. This phenomenon is a crucial part in GA. Mutation probability setting was used to sustain genetic diversity from one generation to the next generation of a chromosome’s population. From its initial solution, mutation will modify either single or more gene rates in a chromosome. The totally new gene rates can be produced after modifying the previous gene rates in the chromosome. The new gene rates chromosome will be added in the gene pool. The GA is able to find better solution compared to the former solution using the new generated gene rate.

### 4.6. Evaluation of the Fitness

In the population, the fitness (quality) of respective chromosome will be analysed in this phase. Each gene has a probability to be found in the gene named as fitness function. For instance, in the population, the fitness function *f*(*x*) is assessed for the respective chromosome *x*. Then, the greatest gene was selected in the selection phase and the other less fit genes will be omitted using the fitness value of quality result. The process is repeated to find the greatest fitness which is the optimum solution of a chromosome as has been determined. 

## 5. Results and Discussions

### 5.1. Simulation Results

DOE results (constructed using FFD and supplemented with face centered CCD) for straight drilled and MGSS cooling channels are presented in Table 3. Based on the results, values of warpage are tabulated for each series of parameters specification in the DOE. The identified variable parameters setting for each series have been simulated via AMI 2013 software. The maximum warpage condition for both straight drilled and MGSS cooling channels are 0.305 mm and 0.160 mm respectively, and the minimum warpage condition are 0.150 mm and 0.090 mm respectively. This shows that the warpage and cooling time for MGSS cooling channels have beed improved even though the optimisation has not yet been applied. MGSS cooling channels contribute to faster heat transfer due to the cooling channels design which follows the shape of the moulded part. In addition, its also provides better thermal distribution that allows the improvement of the warpage condition [15].

### 5.2. Analysis of Variance Result

**Figure 6:**
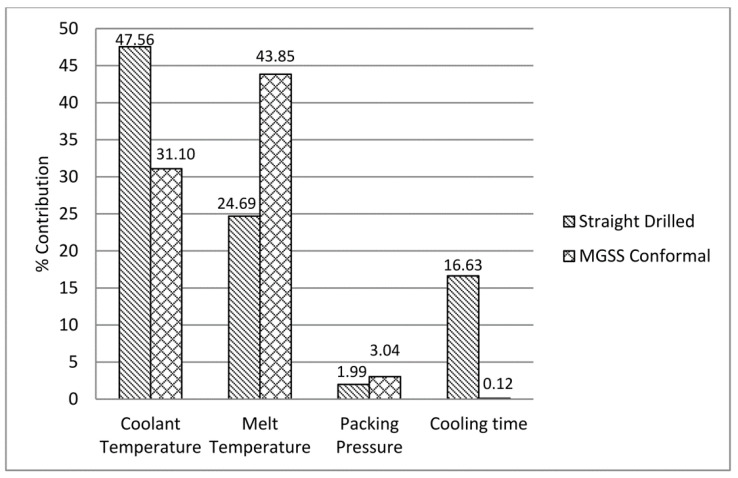
Parameters contribution percentage against warpage for straight drilled and MGSS conformal cooling channels.

After warpage condition has been determined for each DOE run via simulation analysis, ANOVA for FFD was performed for both cases. For straight drilled cooling channels, ANOVA output shows that coolant temperature is the main influencing parameter towards warpage condition, as presented in Figure 6. This finding is consistent with previous researchers who discovered that the same significant factor affects the state of the warpage [27]. The next influencing parameters are melting temperature and the cooling time. These findings are due to uneven shrinkage because the uniformity of heat distribution is low in the mould insert which resulted in the uneven cooling conditions [28]. As stated by Subramanian et al. [29], the temperature variance in core and cavity surfaces affect shrinkage condition. In addition, temperature variation that exists across the wall of moulded parts causes each point to solidify at different times, which consequently lead to higher shrinkage. This effect is more significant compared to the temperature variations of the core and the cavity mould inserts [30]. This phenomenon will lead to either warpage or residual stress. Nevertheless, these conditions also depend on the mechanical robustness of the moulded part design as well. Packing pressure was found to be an insignificant parameter influencing warpage. This indicates that the packing pressure range suggested by the simulation analysis is sufficient in giving a specific amount of material volume [31].

For MGSS cooling channels, ANOVA output defines melting temperature as the main influencing parameter affecting warpage condition. This finding is consistent with previous studies on straight drilled cooling channels, where melting temperature was found to have significant effect on flowrate and curing time of molten plastic which affect the warpage condition [32,33,34]. The next influencing parameter is coolant temperature. Both packing pressure and cooling time indicated less influence towards warpage condition, as presented in Figure 6. 

Furthermore, these findings have proven that the application of MGSS conformal cooling channels would provide better thermal distribution in the mould. This can be justified where coolant temperature was a less influencing parameter towards warpage condition, similar to the straight drilled cooling channels. This is caused by the design strategy of MGSS conformal cooling channels, which aided the moulded part to cool and shrink evenly.

### 5.3. RSM Regression Analysis Result

After defining the significant factors affecting warpage, RSM regression analysis via face centered CCD was performed. Based on the ANOVA results, the calculated F value was greater than the tabulated F value in both cases and this condition indicates that the mathematical models obtained via regression analysis were noteworthy [35]. 

According to the regression analysis via Design Expert 7 software, coefficient of determination, R^2^ that corresponds to the model was 0.9755 as shown in Table 4, while the adjusted coefficient of determination was 0.9662, which indicates that the regression model for straight drilled cooling channels was notable [36,37]. 

On the other hand, the regression analysis for the MGSS cooling channels showed that the coefficient of determination R^2^ corresponding to the model was 0.9431, while, the adjusted coefficient of determination was 0.9249, as shown in Table 5. The variance in the coefficient of determination between both cooling channels is due to the irregular fluctuation of the warpage results, where MGSS cooling channels showed a greater variation associated with straight drilled cooling channels [36,37].

These regression models for warpage toward related parameters have been established for both cooling channels as expressed in Equations (5) and (6) respectively (*A*-coolant temperature; *B*-melting temperature; *C*-packing pressure, and *D*-cooling time). These mathematical model functions (Equations (5) and (6)) are utilized in GSO and GA optimisation methods as the fitness and objective functions.

(5) $$Straight\;Drilled\;Warpage=-0.43-\left(5.323\times 10^{-3}A\right)+\left(0.039B\right)+\left(4.012\times 10^{-3}C\right)\;\;\;\;\;\;\;\;\;\;\;\;\;\;\;\;\;\;\;\;\;\;\;\;\;\;\;\;\;\;\;\;\;\;\;\;\;\;\;\;\;\;\;\;\;\;\;\;\;\;\;\;\;\;\;\;\;\;\;\;\;\;\;\;\;\;\;\;\;\;-\left(7.03\times 10^{-3}D\right)+\left(4.694\times 10^{-5}AC\right)+\left(5.417\times 10^{-5}AD\right)\;\;\;\;\;\;\;\;\;\;\;\;\;\;\;\;\;\;\;\;\;\;\;\;\;\;\;\;\;\;\;\;\;\;\;\;\;\;\;\;\;\;\;\;\;\;\;\;\;\;\;\;\;\;\;\;\;\;\;\;\;\;\;\;\;\;\;\;\;\;\;\;\;\;\;\;\;\;\;\;\;\;\;\;\;\;\;\;\;\;\;\;\;\;\;\;\;\;\;\;\;\;\;\;\;\;-\left(2.414\times 10^{-5}BC\right)-\left(7.77\times 10^{-5}B^{2}\right)$$

(6) $$MGSS\;Warpage=-4.389-\left(2.137\times 10^{-3}A\right)+\left(0.039B\right)-\left(2.586\times 10^{-3}C\right)+\left(3.219\times 10^{-5}AC\right)\;-\left(1.609\times 10^{-5}BC\right)-\left(8.065\times 10^{-5}B^{2}\right)+\left(5.11\times 10^{-5}C^{2}\right)$$

**Figure 7:**
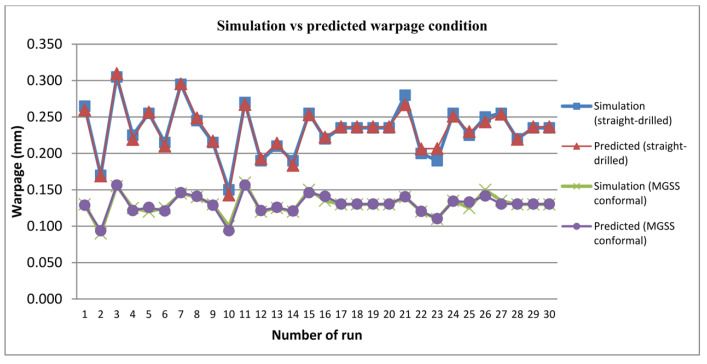
Simulation and predicted results of warpage for both cooling channels.

Furthermore, Equations (5) and (6) were used to determine the warpage prediction values. The results from mathematical response function showed that, these models could predict warpage value with realistic precision for both cooling channels as shown in Figure 7. Moreover, the result showed that warpage was decreased when utilising MGSS conformal cooling channels in each DOE series. 

### 5.4. Optimised Results Based on Simulation Analysis

To obtain the best warpage conditions, the injection moulding processing parameters must be optimally adjusted. Utilising the RSM, lowest warpage value obtained through the polynomial model, with the optimum configuration of parameters yielded the lowest warpage condition. Moreover, the GSO and GA algorithms were executed to define an optimal parameter setting. The algorithm was executed on four selected parameters through the mathematical model gained from RSM in both GSO and GA approaches. Optimised results using RSM, GSO, and GA are summarised and presented in Table 6 and Table 7 for straight drilled and MGSS conformal cooling channels respectively. Based on the results, GA optimisation approach recorded the minimum warpage value compared with other optimisation approaches for both cooling channels, followed by the RSM optimisation approach and finally the GSO approach with respect to the recommended configuration, which was obtained through the simulation analysis for both cooling channels. In addition, MGSS conformal cooling provided less cooling time and better warpage results compared to straight drilled cooling channels due to better thermal distribution in the mould.

### 5.5. Experimental Work Results

**Figure 8:**
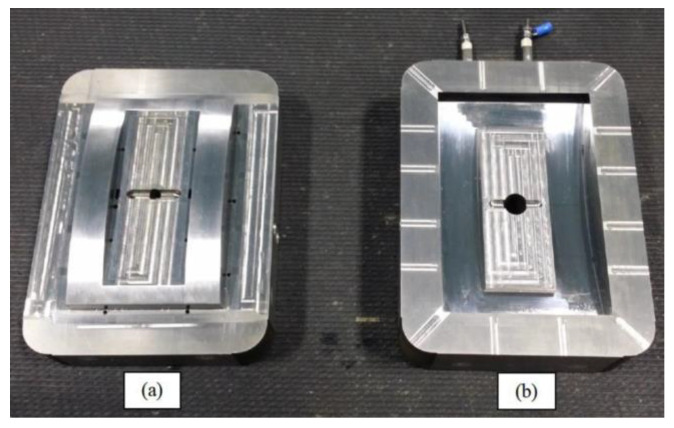
Mould inserts for front panel housing using straight drilled cooling channels, (**a**) core insert, (**b**) cavity insert.

**Figure 9:**
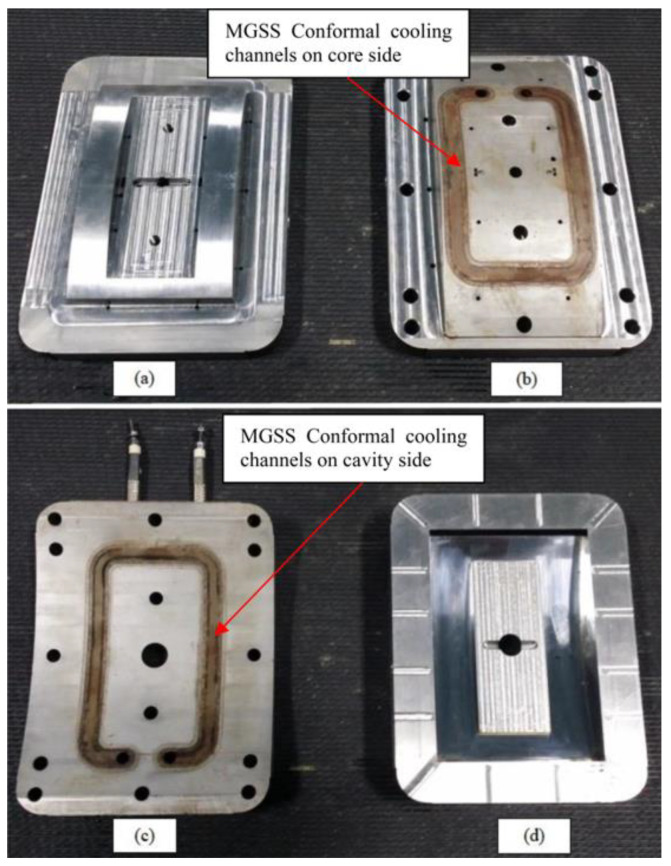
Mould inserts for front panel housing using MGSS conformal cooling channels, (**a**) upper halve core insert, (**b**) lower halve core insert, (**c**) lower halve cavity insert, (**d**) upper halve cavity insert.

Injection moulding of the specimen was conducted experimentally to validate the warpage condition in real situation and was compared to the simulation and optimisation studies. 80 Tonne, Nissei NEX1000 injection moulding machine had been used to mould the specimen by using straight drilled and MGSS conformal cooling channels moulds as presented in Figure 8 and Figure 9 respectively. 

**Figure 10:**
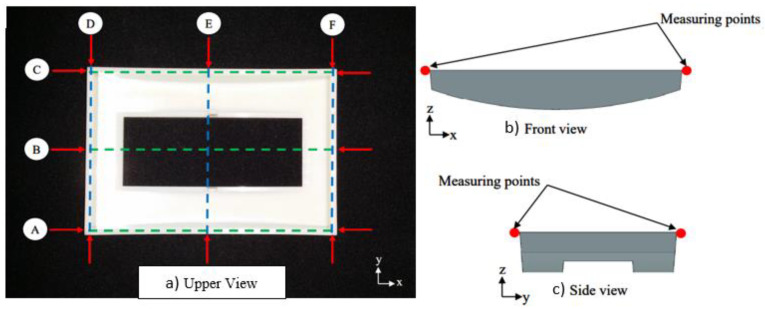
Moulded specimen (front panel housing) with measuring point, (**a**) upper view, (**b**) front view, (**c**) side view.

The specimens were produced using recommended settings from simulation studies for RSM, GSO and GA as shown in Table 6 and Table 7. The warpage on 10 specimens for each recommended parameter setting was measured using Mitutoyo Coordinate Measuring Machine (CMM) after being stored for 48 h at room temperature (21 °C to 25 °C) according to the BS EN ISO 291 standard. In this study, the measuring point as shown in Figure 10 was measured to obtain the warpage condition in x and y directions on each moulded specimens. Equations (7) and (8) as expressed below were utilized to obtain the maximum warpage in x and y directions respectively:

(7) Max. warpage in x direction = Max. value of (A dimension) − (B dimension)or(C dimension) − (B dimension)

(8)                                      Max. warpage in y direction = Max. value of (D dimension) − (E dimension)or(F dimension) − (E dimension)

The results obtained from experimental works were in line with the results obtained from the optimisation work based on simulation studies, where it was proven that the recommended processing parameters setting based on optimisation approaches using RSM, GSO and GA have optimised warpage as compared to the recommended processing parameters setting based on simulation work as presented in Table 6 and Table 7. The warpage condition on the front panel housing from the experimental works using straight drilled and MGSS conformal cooling channels is tabulated in Table 8. However, there is a slight variation in terms of warpage value recorded for simulation and experimental results. This is due to the fact that some parameters in the simulation software have not been considered, for instance, the heat transfer between mould cavity and mould base plate [38]. 

In addition, results from the GA optimisation approach showed better warpage condition for both types of cooling channels as compared to GSO approach, even though both of these optimisation approaches used the same mathematical model generated from RSM. This is because GA is an Evolutionary Intelligence (EI), generates a new chromosomes population which provided a better solution from the previous one by using crossover and mutation operators and assisting GA to jump the discontinuity in the search space and leading to a better exploration compared to the GSO approach [39]. On the other hand, GSO is Swarm Intelligence (SI) based which tends to become inefficient due to high redundancy and has no central control. In addition, GSO algorithm has a few weaknesses such as low accuracy, slow divergence speed and easy to trap into local optimal solution. However, in certain cases, GSO approach might propose a better result compared to GA depending on part design, type of material used and selected variable parameters [40].

## 6. Conclusions

This study aims to improve the quality of moulded parts. The objective of minimizing warpage on the front panel housing was achieved using RSM, GSO and GA optimisation approaches for both straight drilled and conformal cooling channels moulds. The conclusions from the results are as follows:

- The function of the mathematical model to forecast the warpage value with realistic accuracy for both cooling channels can be obtained via RSM.
- For the straight drilled cooling channels mould: ANOVA results showed that the temperature of the coolant is the superior influencing factor towards warpage, followed by the melting temperature and the cooling time due to the uneven spread of heat in the core and cavity inserts.
- For MGSS conformal cooling channels mould: Melting temperature is the most notable factor affecting the warpage on the specimen, followed by coolant temperature, which indicates that the temperature distribution in the core and cavity inserts are more uniform as compared to the straight drilled cooling channels.
- The MGSS conformal cooling channels provide better warpage on the moulded parts either before or after optimisation as compared to the straight drilled cooling channels.
- Based on the experimental results, the configuration setting recommended by GA provides the most optimised warping compared to other optimisation approaches, which had reduced warpage by 32.5% (from 0.375 mm to 0.253 mm) for straight drilled cooling channels and 22.9% (from 0.205 mm to 0.158 mm) for MGSS conformal cooling channels, followed by the RSM optimisation approach, which has reduced warpage by 30.7% (from 0.375 mm to 0.260 mm) for straight drilled cooling channels and by 22% (from 0.205 mm to 0.160 mm) for MGSS conformal cooling channels. Lastly, the GSO approach had reduced warpage by 18.6% (from 0.375 mm to 0.305 mm) for straight drilled cooling channels and 7.3% (from 0.205 mm to 0.190 mm) for MGSS conformal cooling channels.

Nonetheless, the results of the experimental works agree with the results of the optimisation works, which are based on simulation studies, whereby conformal cooling channels with an optimisation approach (in particular the GA optimisation approach) can provide the best quality and productivity of moulded parts. 

## Figures and Tables

**Table 1:** Glowworm Swarm Optimisation (GSO) and Genetic Algorithm (GA) comparison.

Item	Optimisation Algorithm	
	Glowworm Swarm Optimisation, GSO	Genetic Algorithm, GA
Year of develop	2005	1975
Optimisation approach	Swarm Intelligent	Evolutionary Algorithm
Purpose	Find the local finest solution.	Find the best among others

**Table 2:** Variable parameters and levels.

Type of Cooling Channels	Factors	Level	
		Minimum	Maximum
Both	Coolant temperature, A (°C)	25	65
Both	Melt temperature, B (°C)	220	240
Both	Packing pressure, C (MPa)	39.2	62.5
Straight drilled cooling channels	Cooling time, D (s)	22.5	30
MGSS conformal cooling channels	Cooling time, D (s)	9.5	12.5

**Table 3:** Design of experiment (DOE) results for straight drilled and MGSS conformal cooling channels.

Standard Order	Data Source	Variable Parameters for Injection Moulding Simulation					Response	
		Coolant Temperature (°C)	Melt Temperature (°C)	Packing Pressure (MPa)	Cooling Time for Straight Drilled (s)	Cooling Time for MGSS Conformal (s)	Warpage for Straight Drilled (mm)	Warpage for MGSS Conformal (mm)
1	DOE	25	220	39.20	22.50	9.5	0.265	0.130
2		65	220	39.20	22.50	9.5	0.170	0.090
3		25	240	39.20	22.50	9.5	0.305	0.155
4		65	240	39.20	22.50	9.5	0.225	0.125
5		25	220	62.50	22.50	9.5	0.255	0.120
6		65	220	62.50	22.50	9.5	0.215	0.125
7		25	240	62.50	22.50	9.5	0.295	0.145
8		65	240	62.50	22.50	9.5	0.245	0.140
9		25	220	39.20	30.00	12.5	0.215	0.130
10		65	220	39.20	30.00	12.5	0.150	0.100
11		25	240	39.20	30.00	12.5	0.270	0.160
12		65	240	39.20	30.00	12.5	0.190	0.120
13		25	220	62.50	30.00	12.5	0.210	0.125
14		65	220	62.50	30.00	12.5	0.190	0.120
15		25	240	62.50	30.00	12.5	0.255	0.150
16		65	240	62.50	30.00	12.5	0.220	0.135
17	Centre	45	230	50.85	26.25	11.0	0.235	0.130
18		45	230	50.85	26.25	11.0	0.235	0.130
19		45	230	50.85	26.25	11.0	0.235	0.130
20		45	230	50.85	26.25	11.0	0.235	0.130
21	Axial	25	230	50.85	26.25	11.0	0.280	0.140
22		65	230	50.85	26.25	11.0	0.200	0.120
23		45	220	50.85	26.25	11.0	0.190	0.110
24		45	240	50.85	26.25	11.0	0.255	0.135
25		45	230	39.20	26.25	11.0	0.225	0.125
26		45	230	62.50	26.25	11.0	0.250	0.150
27		45	230	50.85	22.50	9.5	0.255	0.135
28		45	230	50.85	30.00	12.5	0.220	0.130
29		45	230	50.85	26.25	11.0	0.235	0.130
30		45	230	50.85	26.25	11.0	0.235	0.130

**Table 4:** ANOVA of response surface model for straight drilled cooling channels.

	Sum of Squares	Degree of Freedom, df	Mean Square	F-Value (Calculated)	Coefficient of Determination, R^2^	Adjusted Coefficient of Determination, RA^2^	F-Value (Tabulated)
Sum of Square Regression, SRR	0.0343	8	0.0043	104.5296	0.9755	0.9662	2.4205
Sum of Square Error, SSE	0.0009	21	4.098 × 10^−5^				
Total	0.0351	29					

**Table 5:** ANOVA of response surface model for MGSS conformal cooling channels.

	Sum of Squares	Degree of Freedom, df	Mean Square	F-Value (Calculated)	Coefficient of Determination, R^2^	Adjusted Coefficient of Determination, RA^2^	F-Value (Tabulated)
Sum of Square Regression, SRR	0.0059	7	0.0008	52.0447	0.9431	0.9249	2.4638
Sum of Square Error, SSE	0.0004	22	1.611 × 10^−5^				
Total	0.0062	29					

**Table 6:** Recommended simulation results versus optimised results using Response Surface Methodology (RSM), GSO and GA for straight drilled cooling channels.

Factors	Recommended Simulation Results	RSM Optimised Results	GSO Optimised Results	GA Optimised Results
Coolant temperature, A (°C)	45.00	64.90	63.60	64.99
Melt temperature, B (°C)	230.00	220.49	220.69	220.00
Packing pressure, C (MPa)	50.61	40.78	45.20	39.20
Cooling time, D (s)	26.92	29.95	25.03	30.00
Warpage, (mm)	0.240	0.148	0.176	0.143
Improvement percentage (%)		38.33	26.67	40.42

**Table 7:** Recommended simulation results versus optimised results using RSM, GSO and GA for MGSS conformal cooling channels.

Factors	Recommended Simulation Results	RSM Optimised Results	GSO Optimised Results	GA Optimised Results
Coolant temperature, A (°C)	45.00	65.00	63.52	65.00
Melt temperature, B (°C)	230.00	220.00	220.61	219.89
Packing pressure, C (Mpa)	50.61	39.45	54.57	39.23
Cooling time, D (s)	11.95	10.95	12.35	12.50
Warpage, (mm)	0.130	0.094	0.108	0.0936
Improvement percentage (%)		27.69	16.92	28

**Table 8:** Average warpage results on the front panel housing based on experimental works for both straight drilled and MGSS conformal cooling channels.

	Simulation Recommended Setting	RSM Recommended Setting	GSO Recommended Setting	GA Recommended Setting
Average warpage result for straight drilled cooling channels (mm)	0.375	0.260	0.305	0.253
Average warpage result for MGSS conformal cooling channels (mm)	0.205	0.160	0.190	0.158
Improvement percentage using MGSS conformal cooling channels mould as compared to straight drilled cooling channels mould (%)	45.33	38.46	37.71	37.55

## Data Availability

Not applicable.

## References

[1] Kitayama S., Miyakawa H., Takano M., Aiba S. (2017). Multi-objective optimization of injection molding process parameters for short cycle time and warpage reduction using conformal cooling channel. Int. J. Adv. Manuf. Technol..

[2] Rajalingam S., Vasant P., Khe C.S., Merican Z., Oo Z. (2016). Optimization of injection molding process parameters by response surface methods. J. Inf. Technol. Softw. Eng..

[3] Kim H.K., Sohn J.S., Ryu Y., Kim S.W., Cha S.W. (2019). Warpage reduction of glass fiber reinforced plastic using microcellular foaming process applied injection molding. Polymers.

[4] Ryu Y., Sohn J.S., Yun C.S., Cha S.W. (2020). Shrinkage and Warpage Minimization of Glass-Fiber-Reinforced Polyamide 6 Parts by Microcellular Foam Injection Molding. Polymers.

[5] Annicchiarico D., Alcock J.R. (2014). Review of factors that affect shrinkage of molded part in injection molding. Mater. Manuf. Process..

[6] Mukras S.M., Omar H.M., Al-Mufadi F.A. (2019). Experimental-based multi-objective optimization of injection molding process parameters. Arab. J. Sci. Eng..

[7] Lam Y.C., Zhai L.Y., Tai K., Fok S.C. (2004). An evolutionary approach for cooling system optimization in plastic injection moulding. Int. J. Prod. Res..

[8] Fahmy M.A. (2019). Modeling and optimization of anisotropic viscoelastic porous structures using CQBEM and moving asymptotes algorithm. Arab. J. Sci. Eng..

[9] Chen W.C., Nguyen M.H., Chiu W.H., Chen T.N., Tai P.H. (2016). Optimization of the plastic injection molding process using the Taguchi method, RSM, and hybrid GA-PSO. Int. J. Adv. Manuf. Technol..

[10] Pervez H., Mozumder M.S., Mourad A.H.I. (2016). Optimization of injection molding parameters for HDPE/TiO_2_ nanocomposites fabrication with multiple performance characteristics using the Taguchi method and grey relational analysis. Materials.

[11] Li H., Liu K., Zhao D., Wang M., Li Q., Hou J. (2018). Multi-objective optimizations for microinjection molding process parameters of biodegradable polymer stent. Materials.

[12] Oliaei E., Heidari B.S., Davachi S.M., Bahrami M., Davoodi S., Hejazi I., Seyfi J. (2016). Warpage and shrinkage optimization of injection-molded plastic spoon parts for biodegradable polymers using Taguchi, ANOVA and artificial neural network methods. J. Mater. Sci. Technol..

[13] Zhao J., Cheng G., Ruan S., Li Z. (2015). Multi-objective optimization design of injection molding process parameters based on the improved efficient global optimization algorithm and non-dominated sorting-based genetic algorithm. Int. J. Adv. Manuf. Technol..

[14] Chung C.Y. (2019). Integrated Optimum Layout of Conformal Cooling Channels and Optimal Injection Molding Process Parameters for Optical Lenses. Appl. Sci..

[15] Park H.S., Dang X.P. (2017). Development of a smart plastic injection mold with conformal cooling channels. Procedia Manuf..

[16] Saifullah A., Masood S. Cycle time reduction in injection moulding with conformal cooling channels. Proceedings of the International Conference on Mechanical Engineering (ICME2007).

[17] Hazir E., Ozcan T. (2019). Response surface methodology integrated with desirability function and genetic algorithm approach for the optimization of CNC machining parameters. Arab. J. Sci. Eng..

[18] Oktem H., Erzurumlu T., Kurtaran H. (2005). Application of response surface methodology in the optimization of cutting conditions for surface roughness. J. Mater. Process. Technol..

[19] Ozcelik B., Erzurumlu T. (2005). Determination of effecting dimensional parameters on warpage of thin shell plastic parts using integrated response surface method and genetic algorithm. Int. Commun. Heat Mass Transf..

[20] Abdelhaleem A.M., Abdellah M.Y., Fathi H.I., Dewidar M. (2016). Mechanical properties of ABS embedded with basalt fiber fillers. J. Manuf. Sci. Prod..

[21] Kaipa K.N., Ghose D. (2017). Glowworm Swarm Optimization: Theory, Algorithms, and Applications.

[22] Marinaki M., Marinakis Y. (2016). A Glowworm swarm optimization algorithm for the vehicle routing problem with stochastic demands. Expert Syst. Appl..

[23] Zainal N., Zain A.M., Radzi N.H.M., Othman M.R. (2016). Glowworm swarm optimization (GSO) for optimization of machining parameters. J. Intell. Manuf..

[24] Kumar A., Kumar P.B., Parhi D.R. (2018). Intelligent navigation of humanoids in cluttered environments using regression analysis and genetic algorithm. Arab. J. Sci. Eng..

[25] Najihah S., Shayfull Z., Nasir S., Saad M.S., Rashidi M., Fathullah M., Noriman N. (2016). Analysis of shrinkage on thick plate part using genetic algorithm. MATEC Web Conf..

[26] Koohestani B. (2020). A crossover operator for improving the efficiency of permutation-based genetic algorithms. Expert Syst. Appl..

[27] Chen W.C., Kurniawan D. (2014). Process parameters optimization for multiple quality characteristics in plastic injection molding using Taguchi method, BPNN, GA, and hybrid PSO-GA. Int. J. Precis. Eng. Man..

[28] Lee D., Chen W.A., Huang T.W., Liu S.J. (2013). Factors influencing the warpage in in-mold decoration injection molded composites. Int. Polym. Proc..

[29] Subramanian N.R., Tingyu L., Seng Y.A. (2005). Optimizing warpage analysis for an optical housing. Mechatronics.

[30] Kamal M.R., Lai-Fook R.A., Hernandez-Aguilar J.R. (2002). Residual thermal stresses in injection moldings of thermoplastics: A theoretical and experimental study. Polym. Eng. Sci..

[31] Huang M.C., Tai C.C. (2001). The effective factors in the warpage problem of an injection-molded part with a thin shell feature. J. Mater. Process. Technol..

[32] Oktem H. (2012). Optimum process conditions on shrinkage of an injected-molded part of DVD-ROM cover using Taguchi robust method. Int. J. Adv. Manuf. Technol..

[33] Wang R., Zeng J., Feng X., Xia Y. (2013). Evaluation of effect of plastic injection molding process parameters on shrinkage based on neural network simulation. J. Macromol. Sci. B.

[34] Yin X.H., Yang C., Li X.P. (2015). Simultaneous control of birefringence and warpage for thermoplastic optical lenses fabricated using microinjection molding. Polym. Plast. Technol. Eng..

[35] Livingstone D.J., Salt D.W. (2005). Judging the significance of multiple linear regression models. J. Med. Chem..

[36] Tanyildizi M.S., Ozer D., Elibol M. (2005). Optimization of α-amylase production by Bacillus sp. using response surface methodology. Process Biochem..

[37] Wang Y.H., Feng J.T., Zhang Q., Zhang X. (2008). Optimization of fermentation condition for antibiotic production by Xenorhabdus nematophila with response surface methodology. J. Appl. Microbiol..

[38] Fernandes C., Pontes A.J., Viana J.C., Gaspar-Cunha A. (2018). Modeling and optimization of the injection-molding process: A review. Adv. Polym. Technol..

[39] Branke J. (2012). Evolutionary Optimization in Dynamic Environments.

[40] Tang Z., Zhou Y., Chen X. (2013). An improved glowworm swarm optimization algorithm based on parallel hybrid mutation. International Conference on Intelligent Computing.

